# Modulation of cGAS-STING signaling by PPARα in a mouse model of ischemia-induced retinopathy

**DOI:** 10.1073/pnas.2208934119

**Published:** 2022-11-21

**Authors:** Xiang Ma, Wenjing Wu, Wentao Liang, Yusuke Takahashi, Jiyang Cai, Jian-xing Ma

**Affiliations:** ^a^Department of Physiology, University of Oklahoma Health Sciences Center, Oklahoma City, OK 73104; ^b^Department of Biochemistry, Wake Forest University School of Medicine, Winston-Salem, NC 27157

**Keywords:** cGAS-STING signaling, PPARα, myeloid cells, neovascularization, ischemia-induced retinopathy

## Abstract

In ischemic retinopathy, overactivated retinal myeloid cells are a crucial driving force of pathological angiogenesis and inflammation. The cyclic GMP-AMP synthase (cGAS) and stimulator of interferon genes (STING) signaling are key regulators of inflammation. This study aims to investigate the association of cGAS-STING signaling with ischemic retinopathy and the regulation of its activation. We found that protein levels of cGAS and STING were markedly up-regulated in retinal myeloid cells isolated from mice with oxygen-induced retinopathy (OIR). Knockout of *Sting* and pharmacological inhibition of STING both alleviated retinal neovascularization (NV) and reduced retinal vascular leakage in OIR. Further, *Sting* knockout and STING inhibitor also alleviated leukocyte adhesion to retinal vasculature and infiltration into the retina as well as microglial activation in OIR. These results suggest that cGAS-STING signaling played a pathogenic role in retinal myeloid cell activation and NV in ischemic retinopathy. To identify the regulation of cGAS-STING signaling in OIR, we evaluated the role of transcription factor peroxisome proliferator-activated receptor α (PPARα). The results demonstrated that PPARα was down-regulated in OIR retinas, primarily in myeloid cells. Furthermore, *Pparα* knockout significantly up-regulated cGAS and STING levels in retinal CD11b^+^ cells, while PPARα agonist inhibited cGAS-STING signaling and cytosolic mitochondrial DNA (mtDNA) release, a causative feature for cGAS activation. Knockout of *Sting* ameliorated retinal NV, hyperpermeability, and leukostasis in *Pparα*^−/−^ mice with OIR. These observations suggest that PPARα regulates cGAS-STING signaling, likely through mtDNA release, and thus, is a potential therapeutic target for ischemic retinopathy.

## Significance

Ischemic retinopathy is a leading cause of blindness and has unmet medical needs for novel therapeutic strategies. In this work, we found that the cGAS-STING pathway was activated in the retina with ischemia-induced retinopathy, predominantly in myeloid cells. Disruption of cGAS-STING signaling alleviated retinal neovascularization and inflammation in the OIR model. Toward the mechanism for cGAS-STING activation in the OIR retina, we found that PPARα plays a significant role in suppressing the expression of cGAS and STING in ischemic retinopathy. These observations have revealed an interaction between PPARα and cGAS-STING signaling and have suggested that dysregulation of the cGAS-STING pathway is a pathogenic mechanism for myeloid cell overactivation and retinal neovascularization in ischemic retinopathy.

Pathological angiogenesis in the retina is a common feature of ocular diseases, such as retinopathy of prematurity (ROP) in newborns, diabetic retinopathy(DR) in the working-age population, and age-related macular degeneration in elderly and is the leading cause of irreversible blindness in developed countries (1–3). Vision-threatening complications, such as retinal hemorrhage, macular edema, retinal detachment, and fibrovascular scarring, are commonly observed in people with persistent pathological angiogenesis in the back of the eye (4). Despite the recent success of anti-VEGF therapies, many patients have recurrent retinal neovascularization (NV) or are resistant to the treatment (5–8). Alternative therapeutic strategies remain to be developed to improve the treatments.

Retinal myeloid cells, including resident retinal microglia and infiltrating monocyte-derived macrophages, are considered a crucial driving force of retinal NV (9–13). Under physiological conditions, retinal microglia have an IL-34-dependent niche in the inner plexiform layer and an IL-34-independent niche in the outer plexiform layer (14). In the developing retina, microglia refine the healthy vasculature, contribute to neurogenesis, and surveil the surrounding microenvironment (15, 16). In response to angiogenic stress such as ischemia, retinal myeloid cells switch from surveillance status to activated forms (10). Activated microglia and macrophages accumulate in the neovascular tufts and guide the pathological neovascular growth, indicating a reciprocal activation of myeloid cells and endothelial cells in the retinal angiogenic niche (10). Retinal microglia have the substantial proliferative capacity and are considered the predominant myeloid cell type in the ischemia-induced retinal NV (9). Accumulating evidence demonstrated the strong plasticity of retinal myeloid cells, which can acquire both the classically activated pro-inflammatory state (M1) and the alternatively activated anti-inflammatory and the pro-angiogenic state (M2) in response to ischemic stimuli (10). However, the molecular mechanisms underlying the activation of microglia/macrophages in pathological angiogenesis remain largely unclear. One of the candidate pathways that can respond to tissue injury under stress conditions is the cyclic GMP-AMP synthase (cGAS) and stimulator of interferon genes (STING) signaling pathway.

cGAS and STING are major regulators of the inflammatory responses to pathogens (17). cGAS recognizes and is activated by double-stranded DNA in the cytoplasm (18). Activated cGAS generates cyclic dinucleotide 2′,3′-cyclic GMP-AMP (cGAMP) from guanosine triphosphate (GTP) and adenosine triphosphate (ATP). As a second messenger, cGAMP binds to and activates the membrane-anchored STING at the endoplasmic reticulum (18). Activated STING initiates a cascade of downstream pathway activation, including those mediated by TRAF-associated NF-κB activator (TANK)-bind kinase 1/interferon regulatory factor 3, NF-κB, and Janus Kinase-signal transducer and activator of transcription (18). Collectively, these signaling events transcriptionally up-regulate cytokines and growth factors, such as interleukin 1β (IL-1β), tumor necrosis factor α (TNF-α), and granulocyte-macrophage colony-stimulating factor, eliciting innate immune responses against pathogen infection (19, 20). In recent years, it has been recognized that the cGAS-STING pathway is also involved in sterile inflammation. Its overactivation, especially in myeloid cells, can cause tissue immunopathology and cell senescence (17).

In ischemic stroke, blockage of the cGAS-STING pathway in microglia attenuated brain inflammation and improved clinical outcomes by promoting the emergence of reparative macrophages (21, 22). The amount of cGAS protein is increased in the retinal pigment epithelium (RPE) of the eyes with geographic atrophy, and cGAS is required for Alu-RNA-induced mitochondrial injury and RPE cell death (23). The gene expression of cGAS and STING in the RPE is subjected to epigenetic regulation (24). In cultured microvascular endothelial cells, transfection of mitochondrial DNA (mtDNA) activated STING and its downstream pathway (25).

The roles of the cGAS-STING pathway in ocular pathological angiogenesis have not been defined. Extensive blood vessel remodeling and formation occur, leading to pathological retinal NV in oxygen-induced retinopathy (OIR) (26, 27). We hypothesize that ischemia stress may activate cGAS-STING signaling in myeloid cells to promote vascular remodeling. In this study, we used genetic and pharmacological approaches to examine the role of the cGAS-STING pathway and its regulation in ischemic retinopathy in a mouse model of OIR. Our results suggest that overactivation of cGAS-STING signaling in retinal myeloid cells plays a pathogenic role in ischemic retinopathy.

## Results

### cGAS-STING Signaling Was Activated in the Retinas of OIR Mice.

**Fig. 1.**
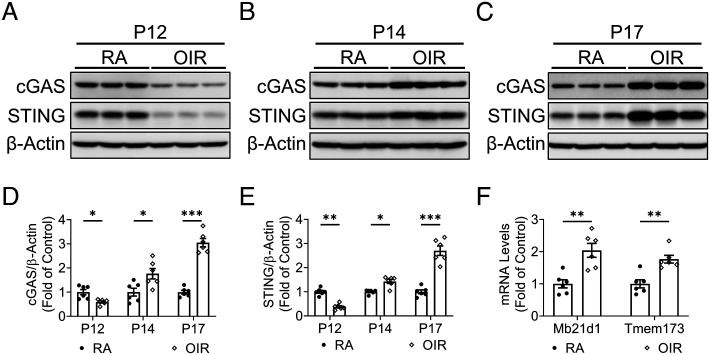
cGAS-STING signaling was up-regulated in the retinas of the OIR model. *A*–*C*: Representative western blots of cGAS and STING in the retinas of OIR mice and RA controls at P12 (*A*), P14 (*B*), and P17 (*C*). *D* and *E*: Protein levels of cGAS and STING in (*A*–*C*) were quantified by densitometry and normalized by β-actin levels (n = 6). *F*: mRNA levels of cGAS (*Mb21d1*) and STING (*Tmem173*) were measured in the retinas of OIR mice and controls at P14 (n = 6). Data were presented as mean ± SEM. *P < 0.05, **P < 0.01, ***P < 0.001.

OIR is associated with an active pro-inflammatory status in the retina (28). We measured time-dependent changes in protein levels of cGAS and STING in the retina of OIR mice (Fig. 1). Compared to age and body weight-matched control mice that were maintained at room air (RA), cGAS and STING showed decreased levels in the OIR retina at P12, immediately following the hyperoxia exposure (Fig. 1*A*). Two days after returning to RA (P14), which was during the relative hypoxia stage, the retinas of OIR mice had elevated cGAS and STING levels relative to RA littermates (Fig. 1*B*). At P17, the day of maximal retinal NV, protein levels of cGAS and STING were markedly increased in the retinas of OIR mice relative to age-matched RA controls (Fig. 1 *C–E*). In addition to the increased protein levels, the mRNA expression of cGAS (*Mb21d1*) and STING (*Tmem173*) was also up-regulated during the ischemic stage (Fig. 1*F*).

### cGAS-STING Signaling Was Overactivated in Retinal Myeloid Cells in OIR Mice.

**Fig. 2.**
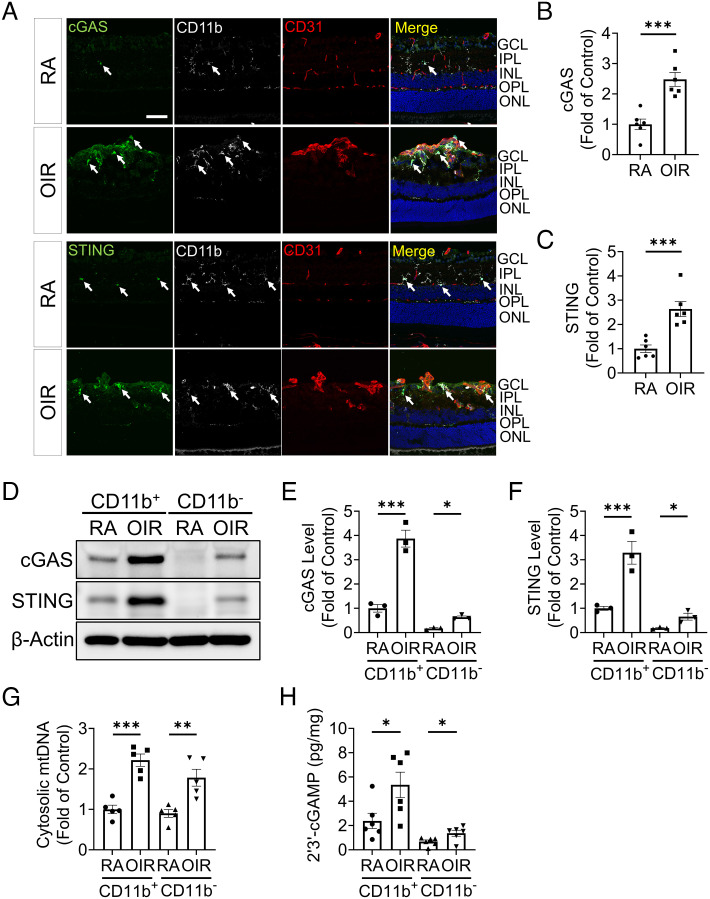
cGAS-STING signaling was overactivated in retinal myeloid cells in the OIR model. *A*: Representative images of immunostaining for cGAS, STING, CD31, and CD11b in retinal cryosections of room air (RA) controls and OIR mice at P17. The white arrows indicated the cGAS and STING staining co-localized with CD11b staining. GCL: ganglion cell layer, IPL: inner plexiform layer, INL: inner nuclear layer, ONL: outer nuclear layer, and OPL: outer plexiform layer. (Scale bar: 50 µm.) *B* and *C*: cGAS and STING immunostaining intensities in (*A*) were quantified by ImageJ software (n = 6). *D*: Representative western blots of cGAS and STING in myeloid cells (CD11b^+^) and non-myeloid cells (CD11b^−^) isolated from RA and OIR retinas at P17 using MACS separation. *E* and *F*: Protein levels of cGAS (*E*) and STING (*F*) in (*D*) were quantified by densitometry and normalized by β-actin levels (n = 3). *G*: Cytosolic mtDNA levels in isolated CD11b^+^cells were quantified by qPCR analysis using primers for a mitochondrial gene *mt-Co1* (n = 5). *H*: Levels of 2′3′-cGAMP were measured in the CD11b^+^ and CD11b^−^ cells of RA and OIR retinas at P17 using 2′3′-cGAMP ELISA kit (n = 6). Data were presented as mean ± SEM. *P < 0.05, **P < 0.01, ***P < 0.001.

Considering the pivotal role of cGAS-STING signaling in innate immune response (29), we determined cGAS and STING expression in retinal myeloid cells (CD11b^+^) of the OIR model. In RA controls at P17, cGAS and STING were mostly expressed in the cell bodies of CD11b^+^ cells distributing in the inner and outer plexiform layers (Fig. 2*A*). In OIR retinas, activated myeloid cells migrated toward preretinal neovascular tufts (CD31^+^) (Fig. 2*A*). The fluorescence intensities of cGAS and STING staining were markedly increased in CD11b^+^ cells near neovascular tufts, suggesting that cGAS-STING signaling was predominantly increased in retinal myeloid cells of ischemic retinopathy (Fig. 2 *A–C*). CD31^+^ cells adjacent to CD11b^+^ cells in neovascular tufts showed mild increases of cGAS and STING staining (Fig. 2 *A–C*). In addition to immunostaining, we measured protein levels of cGAS and STING in MACS-isolated CD11b^+^ cells from the retina. Western blot analysis showed that CD11b^+^ cells had substantially higher cGAS and STING levels than CD11b^-^ cells and were further up-regulated by OIR relative to RA control (Fig. 2 *D–F*). A previous study showed that hypoxia stress induced mtDNA release into the cytosol, which is a common trigger of the activation of DNA sensor, cGAS (30). We found that both CD11b^+^ and CD11b^−^ cells of OIR retinas showed significant increases of cytosolic mtDNA compared to those of RA retinas (Fig. 2*G*). In addition, we measured 2′3′-cGAMP, the second messenger between cGAS and STING. Levels of 2′3′-cGAMP were mainly increased in CD11b^+^ cells in the OIR model (Fig. 2*H*). Collectively, these results suggest that cGAS-STING signaling was overactivated in retinal myeloid cells in response to OIR.

### Knockout of *Sting* Attenuated Retinal NV and Vascular Leakage Caused by OIR.

**Fig. 3.**
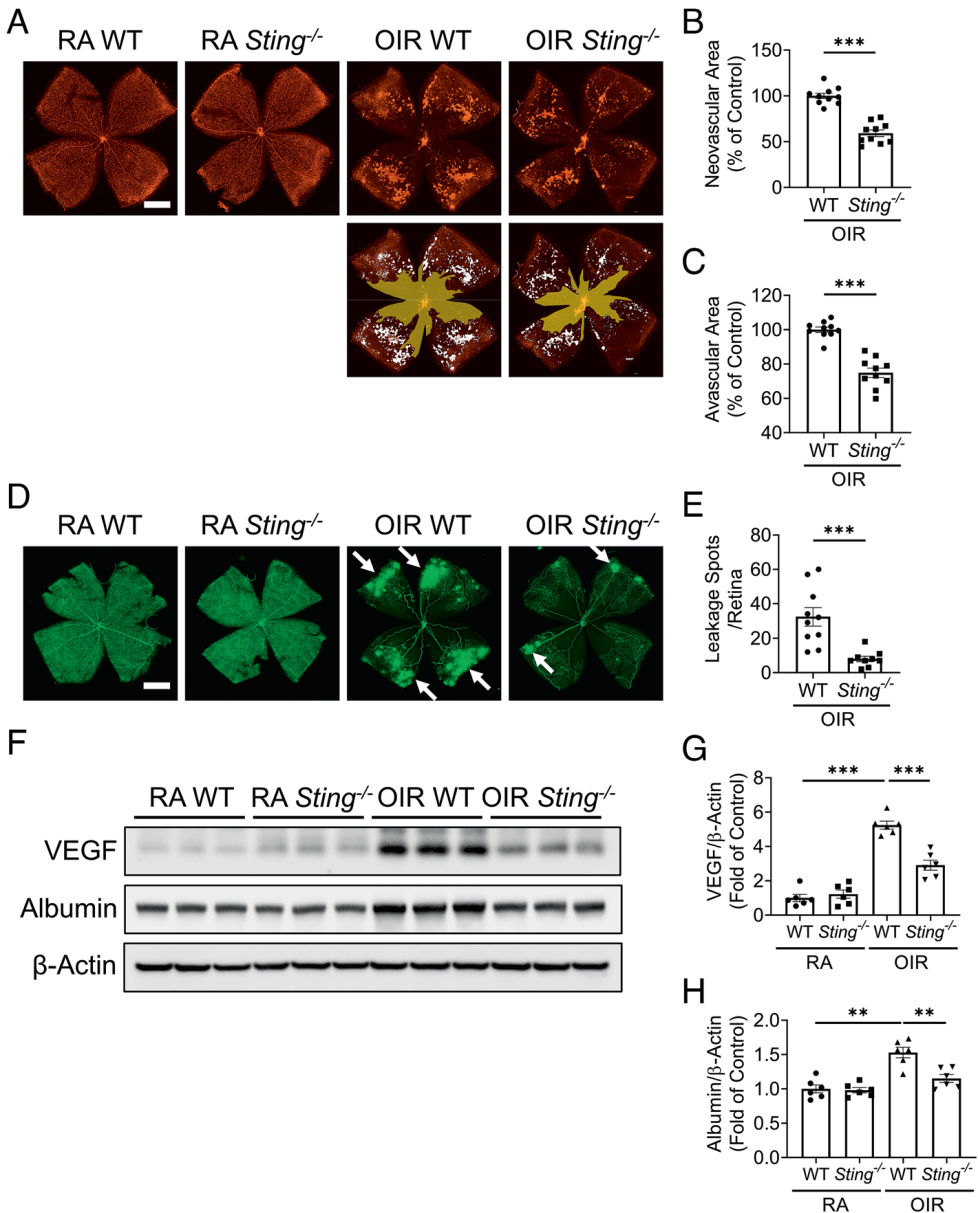
Knockout of *Sting* attenuated NV and vascular leakage in the OIR model. *A*: Representative images of isolectin-stained retinal flat mounts from *Sting^−/−^* mice and WT mice in the RA controls and OIR mice at P17. (Scale bar: 1 mm.) Neovascular areas and avascular areas were quantified and labeled with white color and yellow color, respectively. *B* and *C*: Neovascular areas (*B*) and avascular areas (*C*) in (*A*) were quantified (n = 10). *D*: Representative fluorescein angiography images of retina flat mounts perfused with FITC-dextran (70 kDa). (Scale bar: 1 mm.) The white arrows indicated the spots of vascular leakage. *E*: Numbers of vascular leakage spots in (*D*) were quantified (n = 9–10). *F*: Western blot analysis of VEGF and albumin in the perfused retinas of WT mice and *Sting^−/−^* mice in the OIR and RA groups at P17. *G* and *H*: Protein levels of VEGF (*G*) and albumin (*H*) in (*F*) were quantified by densitometry and normalized by β-actin levels (n = 6). Data were presented as mean ± SEM. **P < 0.01, ***P < 0.001.

To investigate the role of the cGAS-STING in ischemia retinopathy, we compared vaso-obliteration, NV, and vascular leakage in wild-type (WT) and *Sting*^−/−^ mice at P17 (Fig. 3). The physiological retinal vascular development under RA was not influenced by STING deficiency (*SI Appendix*, Fig. S1). On retinal flat mounts, areas of isolectin B4-stained neovascular lesions and vaso-obliterative regions were smaller in OIR *Sting^−/−^* mice compared to OIR WT mice at P17 (Fig. 3 *A*–*C* and *SI Appendix*, Fig. S2). In mice systemically perfused with fluorescein isothiocyanate-dextran (FITC-dextran), *Sting^−/−^* OIR retinas demonstrated much lower vascular permeability and smaller number of leakage spots compared to WT OIR retinas at P17 (Fig. 3 *D* and *E*). On western blots, *Sting^−/−^* OIR mice showed lower levels of vascular endothelial growth factor (VEGF) and albumin in the retina after perfusion relative to WT OIR retinas (Fig. 3 *F*–*H*). VEGF is a key mediator of angiogenesis and vascular leakage; and increased albumin in the perfused retina is an indicator of vascular leakage (10). Taken together, these results suggest that knockout of *Sting* alleviated the retinal NV and vascular leakage in OIR.

### Knockout of *Sting* Attenuated the Aberrant Activation of Retinal Myeloid Cells.

**Fig. 4.**
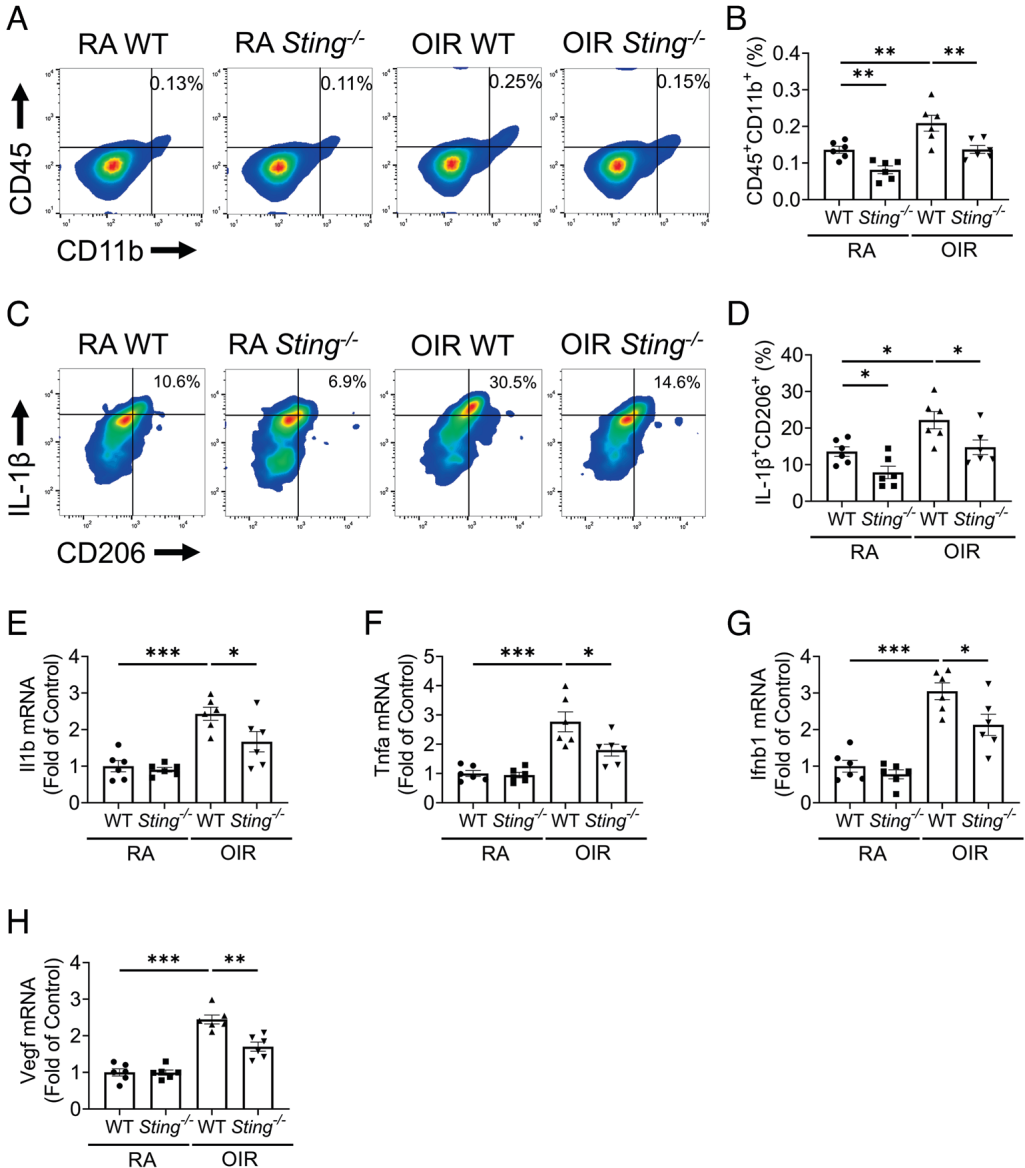
Knockout of *Sting* attenuated the overactivation of retinal myeloid cells in the OIR model. *A*: Representative flow cytometric plots of retinal myeloid cells (CD45^+^CD11b^+^) in the retinas of WT and *Sting^−/−^* mice in RA control and OIR groups at P17. *B*: Flow cytometric quantification of retinal myeloid percentage in the retinal cells in (*A*) (n = 6). *C*: Representative flow cytometric plots of IL-1β^+^CD206^+^ in CD45^+^CD11b^+^ cells in the retinas of RA controls and OIR mice at P17. *D*: Flow cytometric quantification of IL-1β^+^CD206^+^ fractions in retinal CD45^+^CD11b^+^ cells in (*C*) (n = 6). *E*–*H*: qRT-PCR analysis of *Il1b*, *Tnfα*, *Ifnb1*, and *Vegf* mRNA levels in MACS-isolated CD11b^+^ retinal cells at P17 (n = 6). Data were presented as mean ± SEM. *P < 0.05, **P < 0.01, ***P < 0.001.

Overactivation of retinal myeloid cells, including increased cell numbers and their polarized activation toward a mixed M1/M2 state, has been reported in OIR (9, 10). Here, we evaluated the impacts of *Sting* knockout on retinal myeloid activation in OIR using flow cytometry. The gating strategy was presented in *SI Appendix*, Fig. S3. In line with previous reports, we found that the percentage of retinal CD45^+^CD11b^+^ myeloid cells increased in WT OIR retinas compared to WT RA retinas. *Sting*^−/−^ OIR retinas showed significantly fewer myeloid cells compared to WT OIR retinas (Fig. 4 *A* and *B*). In addition, we found that the percentage of IL-1β (M1 marker) and CD206 (M2 marker) double-positive myeloid cells were increased in OIR retinas relative to RA retinas (Fig. 4 *C* and *D*). OIR *Sting^−/−^* mice exhibited lower percentage of IL-1β^+^CD206^+^ myeloid cells in the retina relative to WT OIR mice (Fig. 4 *C* and *D*). Interestingly, *Sting^−/−^* RA retinas showed fewer myeloid cell population and lower percentage of IL-1β^+^CD206^+^ retinal myeloid cells compared to WT RA retinas, suggesting that cGAS-STING pathway is involved in regulating the retinal myeloid cell function at the developmental stage. Furthermore, resident microglia are defined as CD45^low^CD11b^+^ cells in retinal myeloid population (CD45^+^CD11b^+^) (31). In the OIR model, *Sting^−/−^* mice demonstrated a lower number and the percentage of IL-1β^+^CD206^+^ resident microglia relative to WT mice (*SI Appendix*, Fig. S4), indicating that disruption of cGAS-STING signaling suppressed the microglia proliferation and M1/M2 polarization.

We further measured mRNA levels of pro-inflammatory and pro-angiogenic factors in CD11b^+^ retinal cells isolated using MACS. The expression of *Il1b*, *Tnfα*, and interferon 1β (*Ifnb1*) (pro-inflammatory factor), and *Vegf* (pro-angiogenic factor) in CD11b^+^ cells was up-regulated in OIR retinas relative to RA retinas (Fig. 4 *E–H*). In the OIR model, the CD11b^+^ retinal population from *Sting*^−/−^ mice demonstrated lower mRNA levels of *Il1b*, *Tnfα*, *Ifnb1*, and *Vegf* compared to those from WT mice (Fig. 4 *E–H*). Taken together, the data demonstrate that knockout of *Sting* attenuated retinal myeloid cell activation in OIR.

### Knockout of *Sting* Reduced Leukocyte Adhesion and Macrophage Infiltration in the OIR Retinas.

**Fig. 5.**
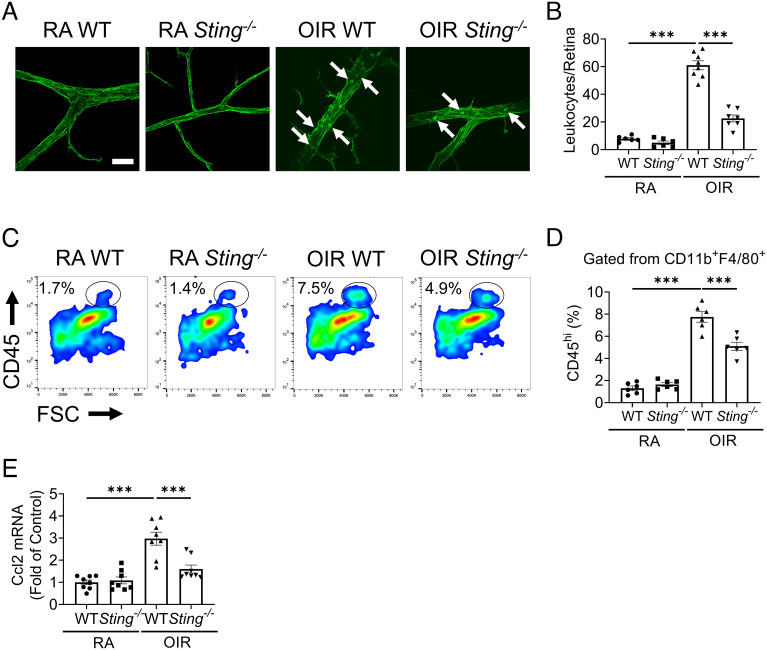
Knockout of *Sting* reduced the number of leukocyte adhesion and macrophage infiltration in the retinas. *A*: Representative images of retinal leukostasis in WT and *Sting*^−/−^ mice in RA control and OIR groups at P17. (Scale bar: 20 µm.) The white arrows indicated the adherent leukocytes. *B*: Quantification of adherent leukocytes in retinal vasculature of RA control and OIR mice in (*A*). *C*: Representative flow cytometric plots of macrophages (CD45^high^ fractions in CD11b^+^F4/80^+^ cells) in the retinas of RA controls and OIR mice at P17. *D*: Quantification of retinal macrophages (CD45^high^ percentage in CD11b^+^F4/80^+^ cells) in (*C*) (n = 6). *E*: qRT-PCR analysis of the *Ccl2* mRNA expression in the retinas of RA controls and OIR mice at P17 (n = 8). Data were presented as mean ± SEM. ***P < 0.001.

As leukocyte adhesion contributes to the development of ischemic retinopathy (32), we measured retinal leukostasis in OIR retinas. WT OIR retinas displayed a robust increase of leukocyte adhesion to retinal vasculature compared to WT RA retinas (Fig. 5 *A* and *B*). *Sting^−/−^* OIR retinas showed a significant decrease of retinal leukostasis relative to WT OIR retinas (Fig. 5 *A* and *B*). Considering that infiltrated monocyte-derived macrophages contribute to the pathological retinal angiogenesis (10), we quantified the CD45^high^CD11b^+^F4/80^+^ macrophages in the OIR retinas. Consistent with the leukostasis result, we observed increased numbers of macrophages in OIR retinas compared to RA controls (Fig. 5 *C* and *D*). Knockout of *Sting* reduced the number of retinal macrophages in the OIR retina, relative to WT OIR retina, indicating an attenuated recruitment of monocytes and macrophages into the retina (Fig. 5 *C* and *D*). We next measured the expression levels of *Ccl2*, a major chemokine involved in the recruitment of macrophages. We found that *Ccl2* mRNA expression was increased in WT OIR retinas compared to WT RA retinas, and *Sting*^−/−^ OIR retinas had lower *Ccl2* mRNA levels than WT OIR retinas (Fig. 5*E*). Thus, knockout of *Sting* attenuated retinal leukostasis and macrophage recruitment in the OIR model.

### STING Inhibitor Attenuated OIR-Induced Retinal Injury.

**Fig. 6.**
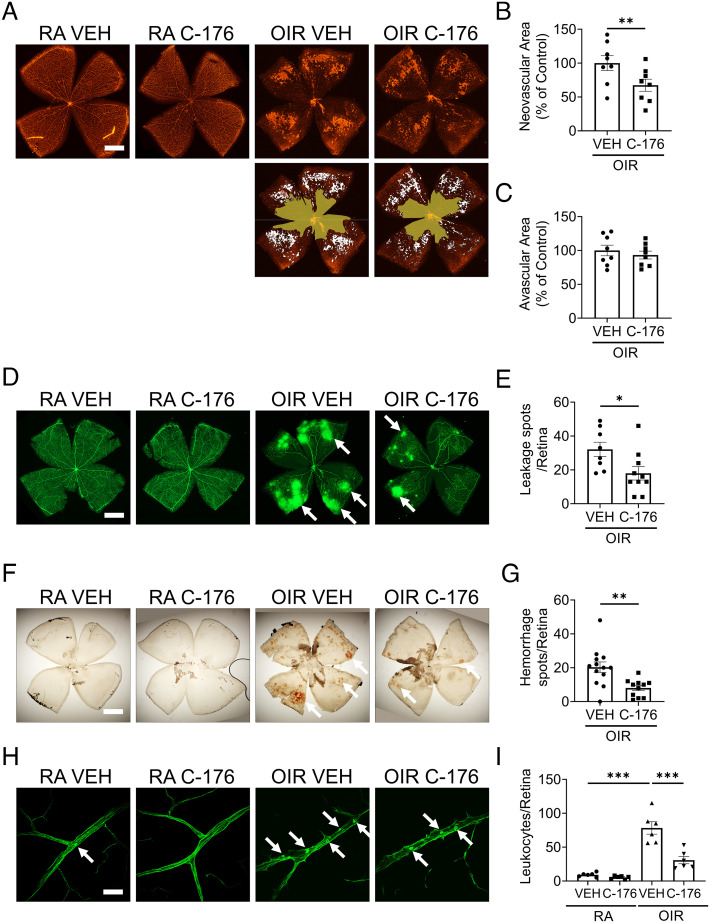
STING inhibitor attenuated retinal NV, vascular leakage, and leukostasis in the OIR model. STING inhibitor C-176 (in 10% DMSO and 90% corn oil) was intraperitoneally injected into RA and OIR mice (21.5 mg/kg/day) from P12 to P17. *A*: Representative images of isolectin-stained retinal flat mounts of vehicle or C-176-treated RA and OIR mice at P17. (Scale bar: 1 mm.) The neovascular areas and avascular areas were labeled with white color and yellow color, respectively. *B* and *C*: The neovascular areas and avascular areas in (*A*) were measured (n = 8). *D*: Representative images of retina flat mounts perfused with FITC-dextran (70 kDa). (Scale bar: 1 mm.) The white arrows indicated the vascular leakage spots. *E*: Vascular leakage spots in (*D*) were quantified (n = 8–10). *F*: Representative images of hemorrhage spots on retinal flat mounts of vehicle or C-176-treated RA controls and OIR mice at P17. (Scale bar: 1 mm.) The white arrows indicate hemorrhage spots. *G*: Quantification of hemorrhage spots in (*F*) (n = 11–13). *H*: Representative images of retinal leukostasis in vehicle or C-176-treated RA control and OIR mice at P17. (Scale bar: 20 µm.) The white arrows indicated the adherent leukocytes. *I*: Quantification of adherent leukocytes in retinal flat mounts of vehicle or C-176-treated RA controls and OIR mice at P17 (n = 6). Data were presented as mean ± SEM. *P < 0.05, **P < 0.01, ***P < 0.001.

In addition to the genetic approach, a mouse STING-specific inhibitor, C-176, was used to determine whether pharmacological inhibition of STING had an impact on the pathologies of OIR, as it allowed us to apply the intervention specifically during the hypoxia stage. WT pups were treated with C-176 at 21.5 mg/kg body weight by daily intraperitoneal injections from P12 to P17. Hemorrhage, leukostasis, and angiogenesis were examined on flat-mounted retinal tissues (Fig. 6). The retinas of OIR mice treated with C-176 exhibited smaller neovascular areas than vehicle-treated controls (Fig. 6 *A* and *B*). There was no significant difference of avascular area between vehicle-treated controls and C-176 group (Fig. 6 *A* and *C*). Fewer spots of FITC-dextran leakage were observed in the C-176 group compared to vehicle-treated controls (Fig. 6 *D* and *E*). Intraretinal hemorrhage is commonly seen in the OIR model. The STING inhibitor significantly reduced the number of hemorrhage spots in the retinas of P17 OIR mice, relative to the vehicle treatment (Fig. 6 *F* and *G*). Moreover, C-176 treatment suppressed the leukocyte adherence relative to vehicle controls in the OIR model (Fig. 6 *H* and *I*).

### PPARα Modulated cGAS-STING Signaling in OIR Retina.

**Fig. 7.**
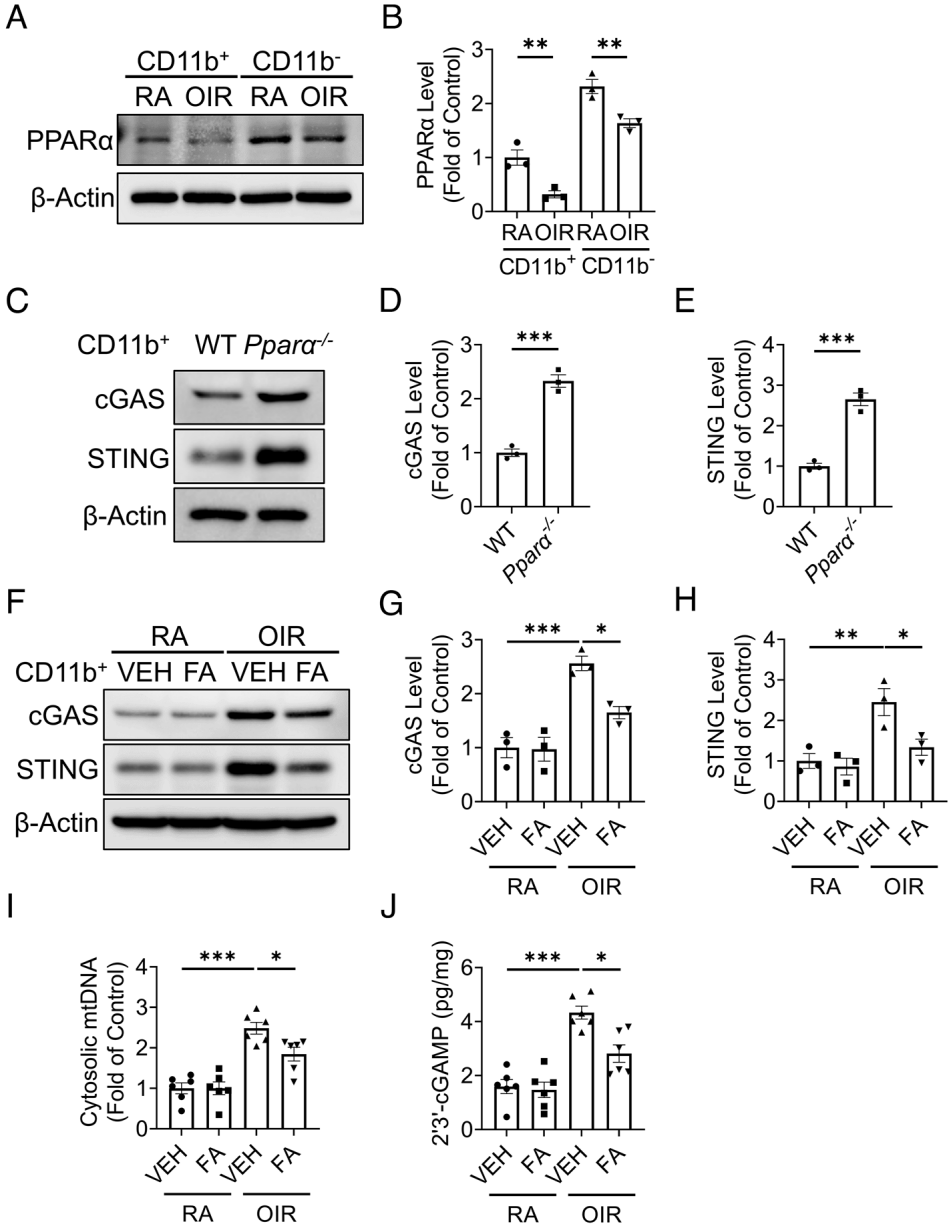
PPARα modulated cGAS-STING signaling in the OIR model. Myeloid cells (CD11b^+^) and non-myeloid cells (CD11b^−^) were isolated from the retinas using the MACS method. *A*: Representative images of western blotting for PPARα in the myeloid cells and non-myeloid cells from the RA and OIR retinas at P17. *B*: Protein levels of PPARα in (*A*) were quantified by densitometry and normalized by β-actin levels (n = 3). *C*: Representative images of western blotting for cGAS and STING in myeloid cells from WT and *Pparα*^−/−^ retinas at P17 at RA. *D* and *E*: Protein levels of cGAS (*D*) and STING (*E*) in (*C*) were quantified by densitometry and normalized by β-actin levels (n = 3). *F*: Representative images of western blotting for cGAS and STING in retinal myeloid cells isolated from RA controls and OIR mice treated with vehicle (VEH) or (FA, 25 mg/kg/day from P12 to P17). *G* and *H*: Protein levels of cGAS (*G*) and STING (*H*) in (*F*) were quantified by densitometry and normalized by β-actin levels (n = 3). *I*: Cytosolic mtDNA levels in retinal myeloid cells isolated from RA controls and OIR mice treated with VEH or FA. Cytosolic mtDNA levels were presented as the fold of VEH-treated RA group (n = 6). *J*: Levels of 2′3′-cGAMP were measured in retinal myeloid cells isolated from RA control and OIR mice treated with VEH or FA at P17 using 2′3′-cGAMP ELISA kit (n = 6). Data were presented as mean ± SEM. *P < 0.05, **P < 0.01, ***P < 0.001.

Our previous work found that peroxisome proliferator-activated receptor α (PPARα), a ligand-activated transcription factor, was down-regulated in whole OIR retinas, contributing to the inflammation in ischemic retinopathy (33). Here, we found that the levels of PPARα were decreased in both CD11b^+^ and CD11b^−^ cells of OIR retinas compared to those in RA retinas (Fig. 7 *A* and *B*). In CD11b^+^ cells isolated from *Pparα*^−/−^ mice at RA, protein levels of cGAS and STING were significantly increased, relative to those from age-matched WT mice (Fig. 7 *C*–*E*). Furthermore, OIR mice treated with PPARα agonist, fenofibric acid (FA), showed lower protein levels of cGAS and STING in CD11b^+^ retinal cells compared to OIR mice with vehicle treatment (Fig. 7 *F–H*). In addition, FA treatment suppressed the increases of cytosolic mtDNA (Fig. 7*I*) and 2′3′-cGAMP (Fig. 7*J*) in CD11b^+^ cells of OIR mice relative to those in vehicle-treated OIR mice. Taken together, these results suggested that PPARα activation suppressed cGAS-STING signaling in myeloid cells of OIR retina.

### Knockout of *Sting* Ameliorated Pathological Angiogenesis in *Pparα*^−/−^ OIR Mice.

**Fig. 8.**
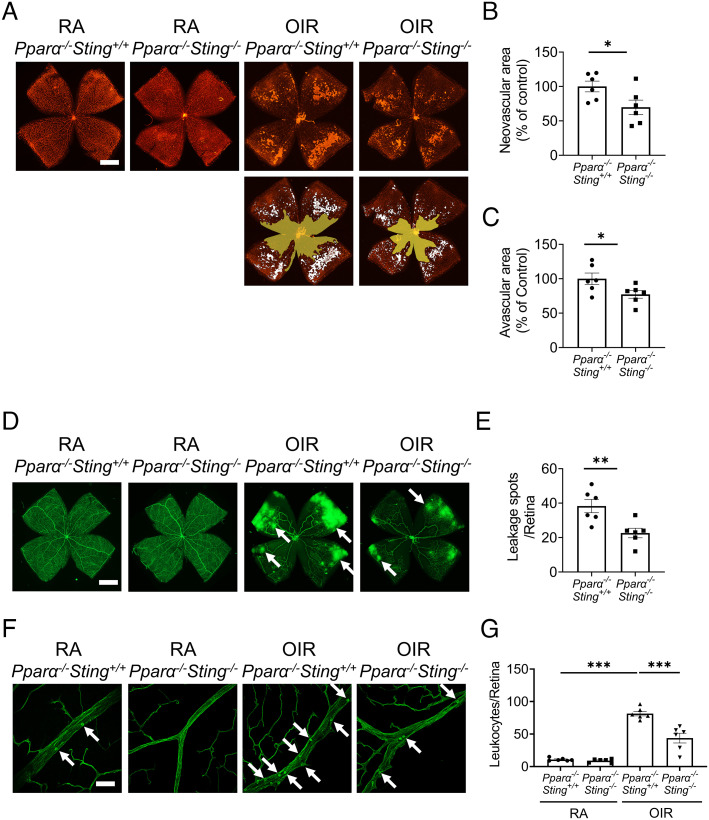
Knockout of *Sting* ameliorated retinal vascular pathologies in *Pparα^−/−^* OIR mice. *A*: Representative images of isolectin-stained retinal flat mounts from *Pparα*^−/−^*Sting*^+/+^ mice and *Pparα^−/−^Sting^−/−^* littermates in RA control and OIR groups at P17. (Scale bar: 1 mm.) The neovascular areas and avascular areas were labeled with white color and yellow color, respectively. *B* and *C*: The quantification of neovascular areas and avascular areas in (*A*) (n = 6). *D*: Representative images of retina flat mounts from *Pparα*^−/−^*Sting*^+/+^ mice and *Pparα^−/−^Sting^−/−^* littermates perfused with FITC-dextran. (Scale bar: 1 mm.) The white arrows indicated the vascular leakage spots. *E*: Vascular leakage spots in (*D*) were quantified (n = 6). *F*: Representative images of retinal leukostasis from *Pparα*^−/−^*Sting*^+/+^ mice and *Pparα^−/−^Sting^−/−^* littermates in RA control and OIR mice at P17. (Scale bar: 20 µm.) The white arrows indicated the adherent leukocytes. *G*: Quantification of adherent leukocytes in retinal flat mounts in (*F*) (n = 6). Data were presented as mean ± SEM. *P < 0.05, **P < 0.01, ***P < 0.001.

Knockout of *Pparα* resulted in significant increases of neovascular areas by 63% and avascular areas by 33% as compared to WT mice at P17 of the OIR model (*SI Appendix*, Fig. S5). Then, we investigated if disruption of cGAS-STING signaling can attenuate vascular pathologies in *Pparα*^−/−^ mice with OIR by generating *Pparα*^−/−^*Sting*^−/−^ mice. Compared to littermate *Pparα*^−/−^*Sting*^+/+^ mice, *Pparα^−/−^Sting^−/−^* mice displayed smaller neovascular areas and avascular areas relative to *Pparα*^−/−^*Sting*^+/+^ mice in the OIR model (Fig. 8 *A–C*). *Pparα^−/−^Sting^−/−^*mice demonstrated a smaller number of vascular leakage spots on flat-mounted retinas and a significant decrease of leukocytes adhered to the retinal vessels compared to *Pparα*^−/−^*Sting*^+/+^ mice with OIR (Fig. 8 *D*–*G*). These results demonstrated that disruption of cGAS-STING signaling ameliorated retinal NV, vascular hyperpermeability, and retinal leukostasis in *Pparα^−/−^* mice with OIR, suggesting that cGAS-STING signaling functions downstream of PPARα in regulating myeloid cell activation in the retina.

## Discussion

The cGAS-STING signaling is a key regulator of the interferon responses against pathogen infection (34). However, its role in pathological retinal angiogenesis has not been well established. The present study demonstrated that the cGAS-STING pathway was activated in the retina with ischemia-induced retinopathy, predominantly in myeloid cells. Genetic knockout and pharmacological inhibition of STING both alleviated retinal NV and myeloid cell activation in the OIR model, suggesting that cGAS-STING signaling played a pathogenic role in ischemia-induced retinopathy. Toward the mechanism for the cGAS-STING activation in the OIR retina, we investigated the role of PPARα, as PPARα plays an important role in maintaining mitochondrial function and integrity (35), and mtDNA release is an activator of cGAS (36). Our results demonstrated that PPARα agonist reduced mitochondrial damage and cytosolic mtDNA release and suppressed cGAS-STING activation and myeloid cell activation in OIR, while *Pparα* knockout alone activated cGAS-STING signaling in retinal myeloid cells. These findings provided evidence that mtDNA release caused by PPARα deficiency contributes to the cGAS-STING activation in ischemic retinopathy. These observations suggested that overactivation of the cGAS-STING pathway is a pathogenic mechanism for myeloid cell activation and retinal NV in ischemic retinopathy and revealed interaction between PPARα and the cGAS-STING pathway.

Myeloid cells are the major players in retinal inflammation (37). Consistent with prior studies (10), myeloid cells in the OIR retina displayed mixed phenotypes of pro-inflammation and pro-angiogenesis (Fig. 4), instead of the dichotomy of M1 or M2 status. Here we found that myeloid cells have a more prominent cGAS-STING activation in OIR. To establish the role of cGAS-STING activation in retinal vascular dysfunction and NV, we used *Sting*^−/−^ mice for OIR and found that ablation of *Sting* ameliorated myeloid cell activation, leukostasis, vascular leakage, and NV in OIR retinas. Similarly, pharmacological inhibition of STING using C-176 also suppressed these OIR pathologies in WT mice. Further, ablation of *Sting* suppressed the expression of pro-inflammatory cytokines such as IL-1β, TNF-α, and IFN-β, as well as the pro-angiogenic factors, including VEGF (Fig. 4). These myeloid cell-derived soluble factors have paracrine functions in the retina and contribute to the pathological angiogenesis. Retinal NV involves significant remodeling (38). Activated myeloid cells can acquire a reparative phenotype and be responsible for the repair and removal of damaged (sub) cellular structures (21). Ischemic stress leads to the exposure of danger-associated molecular patterns (DAMPs), especially double-strand DNA from damaged mitochondria (Fig. 2*G*), resulting in aberrant myeloid activation and delayed inflammation resolution (22). Activated myeloid cells produce cytokines and growth factors to further promote angiogenesis. Inhibiting the cGAS-STING pathway can be an effective way to interrupt this feedback loop and re-establish the homeostasis in the retinal tissue after injury.

Retinal myeloid cells (CD45^+^CD11b^+^) are composed of resident microglia (CD45^low^CD11b^+^) and infiltrating monocyte-derived macrophages (CD45^high^CD11b^+^) (31). A lineage tracing study indicated that resident microglia constitute the predominant population of retinal myeloid cells (>90%) during ischemia and physiological development (9). Under OIR conditions, ischemic stress stimulated the proliferation of resident microglia and M1/M2 hybrid polarization of resident microglia (*SI Appendix*, Fig. S4). Knockout of *Sting* mitigated the overactivation of resident microglia in ischemic retinopathy by suppressing its proliferation and polarization. During retinal development, resident microglia undergo proliferation and transiently express activation markers (9, 39). cGAS-STING signaling is known to modulate the expression of multiple cytokines that have been implicated in microglia proliferation and activation, such as type I interferon and IL-34, an alternative ligand for the colony-stimulating factor-1 receptor (40, 41). This may explain the importance of the cGAS-STING axis in maintaining the number and activation of retinal resident microglia in RA conditions (*SI Appendix*, Fig. S4).

Our study characterized the cGAS-STING signaling in a mouse model of ischemic retinopathy. As shown in Fig. 1, levels of cGAS and STING in the retina were decreased immediately after the hyperoxia stage. This was followed by their marked upregulation during the relative ischemic stage, when the retinal inflammation and NV occurred (31). It has been reported that the high oxygen tension can down-regulate genes involved in multiple signaling pathways related to inflammation, angiogenesis, development, and metabolism (42). The bi-phasic response was observed in other inflammatory regulators, such as HIF-1α and RORα (43). Another interesting finding from our study is that *Sting*^−/−^ mice demonstrated a smaller avascular area than WT mice at P12 and P17 of OIR, while the treatment with STING inhibitor from P12 to P17 had no effect on the avascular area. Such results suggest that cGAS-STING signaling can have an impact on vaso-obliteration during the hyperoxia stage. Considering the interplay between cGAS-STING signaling and cell death pathways (44), cGAS-STING signaling may have the effect on endothelial cell death during vaso-regression. Future study is warranted to further investigate the cellular mechanisms of cGAS-STING signaling-mediated vascular obliteration. Furthermore, blockade of the cGAS-STING pathway inhibited expression of inflammatory factors in myeloid cells of ischemic retina, including TNF-α, IL-1β, and IFN-β (Fig. 4). It is known that in addition to VEGF, inflammatory cytokines, such as IL-1β and TNF-α, play pathogenic roles in NV of ischemic retinopathy (45, 46). An elevation of myeloid IL-1β and TNF-α coincides with the development of pathological angiogenesis in ischemic retinopathy (47, 48). IFN-β was reported to cause retinal vascular inflammation in DR (49). Therefore, modulation of the cGAS-STING signaling pathway in myeloid cells may serve as an approach to treat vascular eye diseases. The STING inhibitors may be applied to ROP patients resistant to anti-VEGF therapies. Anti-STING and anti-VEGF therapies may have synergistic effects in mitigating the retinal inflammation and pathological angiogenesis in ischemic retinopathy.

Another finding of the present study is that PPARα regulates the activation of cGAS-STING signaling in OIR retinas (Fig. 7). PPARα is a ligand-inducible transcription factor that belongs to the nuclear receptor superfamily and plays an important role in lipid metabolism, glucose homeostasis, and mitochondrial integrity and function (50). Our previous work demonstrated that PPARα was down-regulated in the retina of the OIR and diabetic animal models (33, 51). The present study demonstrated that PPARα is expressed at a high level in retinal myeloid cells in RA condition, and its expression is down-regulated in these cells of the OIR model. Furthermore, knockout of *Pparα* alone resulted in upregulation of cGAS and STING levels in retinal myeloid cells. In contrast, activation of PPARα using fenofibric acid suppressed the increases of cGAS and STING levels in the retinal myeloid cells from WT OIR mice, suggesting an inhibitory role of PPARα in cGAS-STING signaling. Moreover, double knockout mice of *Pparα^−/−^Sting^−/−^* showed less severe vascular pathologies than *Pparα*^−/−^ mice in the OIR model (Fig. 8), suggesting that anti-angiogenic effects of PPARα are mediated, at least in part, through the cGAS-STING signaling in OIR.

**Fig. 9.**
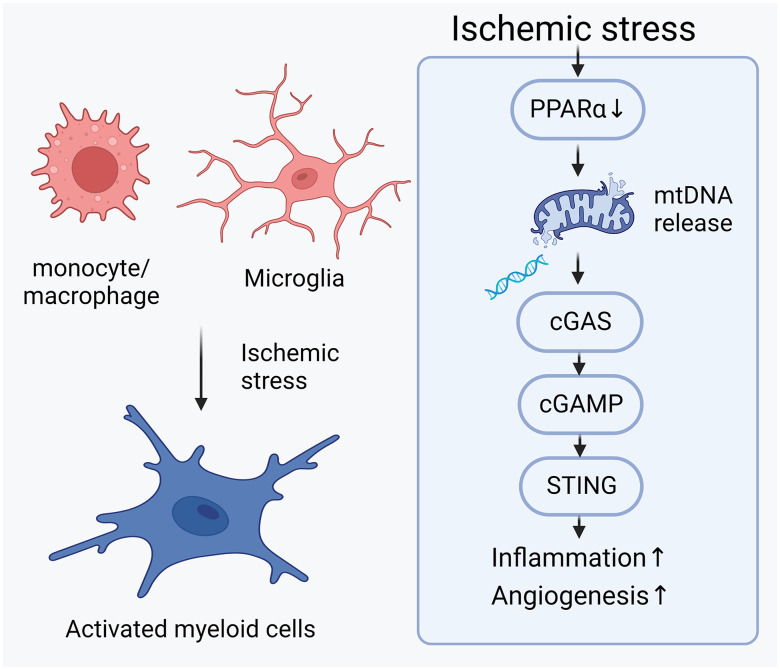
Pathogenic role of cGAS-STING signaling in ischemic retinopathy. Ischemic stress in the retinas transited the myeloid cells (including resident microglia and monocyte/macrophage derived from the bone marrow) from resting form to activated form. Downregulation of PPARα in the ischemic retina contributes to the mitochondrial stress, resulting in the release of mitochondrial DNA (mtDNA) into cytosol. Cytosolic mtDNA activated DNA sensor cGAS, which synthesized the second messenger 2′3′-cGAMP to activated downstream adaptor protein STING. Activation of cGAS-STING signaling in myeloid cells promoted the secretion of pro-inflammatory and pro-angiogenic cytokines, contributing to the inflammation and angiogenesis in ischemic retinas. The schematic figure was generated in BioRender.com.

A recent study reported that PPARα stimulation suppressed interferon production through modulation of reactive oxygen species (ROS) production during infection (52). The interaction between PPARα and the cGAS-STING pathway is previously unknown. MtDNA released into the cytosol is a known activator of cGAS (53). PPARα is known to regulate mitochondrial function and integrity (54). Therefore, we measured mtDNA release in the retina of OIR mice. Our results showed that cytosolic levels of mtDNA are significantly higher in the OIR retina relative to RA control, which can activate the cGAS in myeloid cells (Fig. 9). Treatment of OIR mice with the PPARα agonist fenofibric acid significantly reduced the mtDNA release into the cytosol. Taken together, our results demonstrated an interaction of PPARα with cGAS-STING signaling in retinal myeloid cells, suggesting that downregulation of PPARα in myeloid cells of the OIR retina may result in mitochondrial damage and mtDNA release into the cytosol, which may represent at least one of the mechanisms, for the activation of cGAS-STING signaling, in the OIR retina.

Our study has some limitations. We used global *Sting*^−/−^ mice or global administration of STING inhibitor. To define cell type-specific function, myeloid cell-specific knockout mice will be generated in the future. In current study, we found that cGAS-STING signaling was moderately up-regulated in retinal endothelial cells of ischemic retina. Previous publication showed that cGAS-STING signaling is involved in endothelial inflammation caused by lipid overload or bacterial endotoxin (25, 55). Inflammation is known to mediate angiogenesis in ischemic diseases (56). We cannot rule out the potential pro-angiogenic role of endothelial cGAS-STING signaling in abnormal NV in ischemic retinopathy.

In conclusion, PPARα deficiency in ischemic retinopathy contributes to cGAS-STING activation which plays a key role in myeloid cell activation, retinal inflammation, and NV in OIR. Thus, blockage of the cGAS-STING pathway using PPARα agonist has potential to become a therapeutic strategy for the retinal vascular dysfunction and NV. Retinal myeloid cells may serve as a therapeutic target for pathological angiogenesis.

## Materials and Methods

### Reagents.

Alexa-594-labeled isolectin B4 was purchased from ThermoFisher Scientific (Waltham, MA). An anti-CD11b antibody conjugated with microbeads and LS column were purchased from Miltenyi Biotec (Auburn, CA). FA was purchased from AK Scientific (Union, CA). Fluorescein-labeled Concanavalin A (Con A) was purchased from Vector Laboratories (Burlingame, CA). Fluorescein isothiocyanate-dextran (FITC-dextran, 70 kDa) and collagenase D were obtained from Sigma-Aldrich (St. Louis, MO). N-(4-iodophenyl)-5-nitro-2-furancarboxamide (C-176, a STING inhibitor) and 2′3′-cGAMP enzyme-linked immunosorbent assay (ELISA) kit were obtained from Cayman Chemical Company (Ann Arbor, Michigan). RNeasy mini kits were acquired from QIAGEN (Germantown, MD).

### Animals.

*Pparα*^−/−^ mice in the C57BL/6J background, *Sting*^−/−^ mice in the C57BL/6N background, and WT C57BL/6J mice were obtained from Jackson Laboratory (Bar Harbor, ME). To eliminate the spontaneous *rd8* mutation in 6N background, *Sting*^−/−^ mice were crossbred with C57BL/6J mice to generate knockout mice in the C57BL/6J background for this study. The absence of *rd8* mutation was validated by genotyping (57). All animal experiments followed the protocols approved by the Institute Animal Care and Use Committee at the University of Oklahoma Health Sciences Center. All procedures using experimental animals were performed in compliance with the Use of Animals of Association for Research in Vision and Ophthalmology.

### The Model of OIR.

The OIR model was generated as described previously (58). Neonatal mouse pups were raised in RA of 21% oxygen to postnatal day 7 (P7). Then, mouse pups were exposed to 75% oxygen for 5 d from P7 to P12 (hyperoxic stage with retinal vaso-obliteration). The animals were returned to RA at P12 and maintained in RA for another 5 d from P12 to P17 (relative hypoxic stage with retinal NV). Age-matched pups raised in RA were used as the RA controls. For the treatment of the STING inhibitor, mouse pups were intraperitoneally injected with an inhibitor specific for mouse STING, C-176, from P12 to P17 (19). The dose of C-176 (21.5 mg/kg/day) was used based on a previous publication with similar pathological model pertaining to mtDNA leakage into the cytosol (20). The control group was injected with the vehicle containing 10% DMSO and 90% corn oil. For the treatment of PPARα agonist, mouse pups were intraperitoneally injected with FA (25 mg/kg/day) daily from P12 to P17. The control group was injected with the vehicle (DMSO/corn oil).

### Permeability Assay and Retinal Leukostasis Assay.

Mice at P17 were euthanized and perfused with pre-warmed phosphate-buffered saline (PBS, pH 7.4) containing 50 mg/ml FITC-dextran (permeability assay) or 20 μg/ml Con A (retinal leukostasis assay) via the left ventricle. After fixation in 4% paraformaldehyde, the retinas were flat-mounted and photographed under a fluorescence microscope. The quantification of leakage spots on the FITC-dextran perfused retinal flat mount and the number of adhered leukocytes in the retinal blood vessels were quantified by researchers masked to the animal group information.

### Isolation of Retinal Myeloid Cells.

CD11b^+^ cells were isolated from the retina as described previously (10). Briefly, fresh retinas were digested with collagenase D to generate single cell suspension, which was further incubated with an anti-CD11b antibody conjugated with magnetic MACS beads (Miltenyi Biotec, Cambridge, MA) for 30 min at 4°C. The CD11b^+^ and CD11b^−^ cells were then separated using LS column (Miltenyi Biotec, Cambridge, MA). Isolated cells were collected and analyzed by quantitative reverse transcription PCR (qRT-PCR) or western blot analyses.

### Measurement of Cytosolic mtDNA.

Cytosolic mtDNA levels were quantified as previously described (30, 53). Briefly, cytosolic and nuclear fractions were isolated using the subcellular protein fractionation kit (ThermoFisher, Rockford, IL). The DNA in cytosolic and nuclear fractions was extracted using phenol/chloroform/isoamyl alcohol method. mtDNA in the cytosol was measured by real-time RT-PCR using the primers for a mitochondrial gene, *cytochrome c oxidase 1 (mt-Co1).* Levels of cytosolic mtDNA were normalized to DNA levels of the *β-actin (Actb)* gene in the nuclear fractions. The primer sequences are given in *SI Appendix*, Table S1.

### Detection of Cellular 2′3′-cGAMP.

Cellular levels of 2′3′-cGAMP were measured using a competitive ELISA kit (Cayman, Ann Arbor, Michigan), following manufacture’s guide. Briefly, retinal cells were lysed in M-PER™ mammalian protein extraction reagent (ThermoFisher) and then centrifuged at 12,000 x *g* for 10 min. The supernatant (cytosolic fraction) was used for 2′3′-cGAMP measurement. Levels of 2′3′-cGAMP were normalized by the total protein concentration in the supernatant as measured by bicinchoninic acid (BCA) assay.

### Western Blot Analysis.

Western blot analysis was described previously (59). The retinas were dissected and snap-frozen in liquid nitrogen and stored in −80°C until use. Tissues or cell samples were lysed in the Laemmli sample buffer containing 65 mM Tris–HCl (pH 6.8), 10% glycerol, 2% SDS, and supplemented with 1% proteinase inhibitor cocktail (Sigma, Burlington, MA). After brief sonication, the samples were centrifuged at 12,000 x *g* for 10 min at 4°C. Protein concentrations of the supernatant were determined by BCA assay (ThermoFisher, Waltham, MA). Samples with equal amounts of proteins were resolved in SDS-PAGE gels and transferred to nitrocellulose membranes. After blocking with 10% non-fatty milk in Tris-buffered saline with 0.1% Tween 20 (TBST), the membranes were incubated with primary antibodies overnight at 4°C, followed by incubation with secondary antibodies for 2 h. All antibodies were diluted in TBST containing 5% bovine serum albumin (BSA). Details of primary and secondary antibodies used in this study are given in *SI Appendix*, Table S2.

### Immunofluorescent Staining.

Mouse eyeballs were enucleated and post-fixed in 4% paraformaldehyde in phosphate-buffered saline (PBS, pH 7.4) for 1 h. After sucrose gradient dehydration, the posterior eye cups were embedded in optimal cutting temperature (OCT) compound and sectioned to 8 µm on a Leica cryostat (Buffalo Grove, IL). For immunostaining, the cryosections were blocked with 5% BSA containing 0.2% Triton X-100 for 2 h. The slides were then incubated with primary antibodies overnight at 4°C. After three washes with PBS, the slides were incubated with secondary antibodies for 2 h. The slides were counterstained with 4’,6-diamidino-2-phenylindole (DAPI) and mounted with the Vectashield mounting buffer (Vector Laboratories, Burlingame, CA). The slides were imaged with a Leica SP8 laser scanning confocal microscope (Buffalo Grove, IL). The intensity in retinal sections was quantified using color histogram in Image J software (60).

For whole-mount retinas, retinas were isolated from fixed eyeballs under a dissection microscope. The retinas were incubated with Alexa-594-labeled isolectin B4 (10 μg/ml, Invitrogen, Carlsbad, CA) overnight at 4°C and then flat-mounted. The retinal flat mounts were imaged with a Cytation 1 imaging reader (Agilent BioTek, Santa Clara, CA). The neovascular areas and avascular areas in the retinas were quantified by SWIFT_NV macros in Image J software (60) and Adobe Photoshop according to a documented method (61).

### Flow Cytometry Analysis.

Flow cytometry analysis was performed as described previously (62). Briefly, the isolated retinas from control and OIR mice were digested with 200 µg/ml collagenase D at 37°C for 15 min. The resulted single cell suspension was washed in flow cytometry staining buffer (eBiosciences, San Diego, CA). The Fc receptors were blocked with an anti-mouse CD16/CD32 antibody (1:50) for 15 min. Cell surface markers were stained with fluorophore-conjugated antibodies at 4°C for 30 min. For intracellular staining, the samples were fixed with fixation and permeabilization buffer (Tonbo Biosciences, San Diego, CA) at 4°C for 30 min, followed by incubation with antibodies against intracellular antigens. After staining, samples were analyzed by an Attune NxT flow cytometer (ThermoFisher, Waltham, MA). Details of flow antibodies used in this study are given in *SI Appendix*, Table S2.

### qRT-PCR.

Whole retinas or isolated CD11b^+^ retinal cells were lysed in Trizol reagent (Invitrogen, Waltham, MA). RNA was extracted using RNeasy kit (QIAGEN, Germantown, MD). cDNA was generated with reverse transcriptase reaction. mRNA levels of target genes were measured by qRT-PCR with the primers given in *SI Appendix*, Table S1.

### Statistics Analysis.

At least six mice per group were used for the animal experiments. At least three replicates were performed separately for MACS cell separation from pooled samples. Results were presented as mean ± SEM. Statistical analyses were conducted using the two-tailed Student’s *t* test for comparison of two groups, or using one-way ANOVA followed by the Student–Newman–Keuls test when more than two groups were compared. The *P* value of <0.05 was considered statistically significant.

## Supplementary Material

Appendix 01 (PDF)

## Data Availability

All study data are included in the article and/or *SI Appendix*.
