# Supplementary material for: Modulation of cGAS-STING signaling by PPARα in a mouse model of ischemia-induced retinopathy
Source: Proc Natl Acad Sci U S A. 2022 Nov 21;119(48):e2208934119. doi: 10.1073/pnas.2208934119 (PMC9860285; doi:10.1073/pnas.2208934119)
Supplement: Supplementary file 1 — Appendix 01 (PDF) [file pnas.2208934119.sapp.pdf]

Supporting Information for

Modulation of cGAS-STING Signaling by PPARα in a Mouse Model of Ischemia-Induced Retinopathy

Xiang Ma[1,2], Wenjing Wu[1,2], Wentao Liang[1,2], Yusuke Takahashi[1,2], Jiyang Cai[1*], Jian-xing Ma[1,2*]

[1]Department of Physiology, University of Oklahoma Health Sciences Center, Oklahoma City, OK, USA.

[2]Department of Biochemistry, Wake Forest University School of Medicine, Winston-Salem, NC, USA.

***Correspondence to**

Jiyang Cai, University of Oklahoma Health Sciences Center, BMSB 662, 940 Stanton L. Young Blvd, Oklahoma City, OK 73104

Tel: 405-271-2226; E-mail: Jiyang-cai@ouhsc.edu

Jian-Xing Ma, Department of Biochemistry, Wake Forest University School of Medicine, Winston-Salem, NC, 27157

Tel: +1 336-716-4676; E-mail: jianma@wakehealth.edu

This PDF file includes:

Figures S1 to S5

Tables S1 to S2

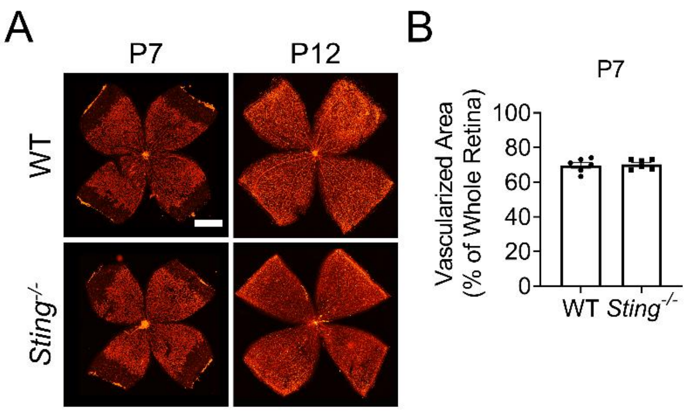

Figure S1

Figure S1. *Sting*$^{-/-}$ mice had normal retinal vascular development. A: Representative images of isolectin-stained retinal flatmounts from WT and *Sting*$^{-/-}$ mice in RA at P7 and P12. Retinal vasculature was expanding toward periphery at P7 and covered whole retinal areas at P12 in both genotypes. Scale bar: 1 mm. B: The percentage of vascularized areas in whole retinas at P7 in (A) were measured (n = 6). Data were presented as mean ± SEM.

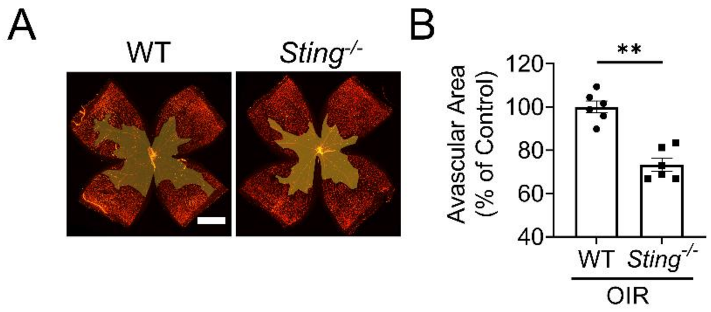

Figure S2

Figure S2. Knockout of *Sting* suppressed the vaso-obliteration at the hyperoxia stage of the OIR model. A: Representative images of isolectin-stained retinal flatmounts from WT and *Sting*$^{-/-}$ mice immediately after hyperoxic stage in the OIR model at P12. Scale bar: 1 mm. Avascular areas were labeled with yellow color. B: Avascular areas in (A) were quantified (n = 6). Data were presented as mean ± SEM. ** P < 0.01.

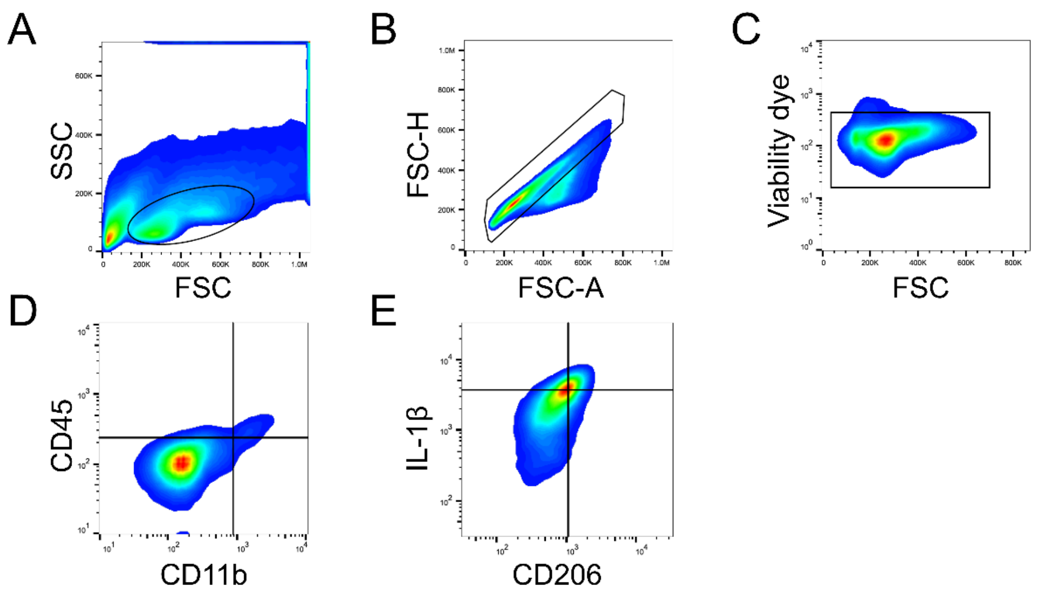

Figure S3

Figure S3. Gating strategy for flow cytometry analysis. A: Retinal cell suspensions were analyzed by flow cytometry. B: Singlets were selected, and multiplets were excluded. C: Dead cells were excluded using fixable viability dye. D: Retinal myeloid cells were defined as $CD45^+CD11b^+$ cells. E: The $IL1\beta^+$ and $CD206^+$ fractions were measured in retinal myeloid cells.

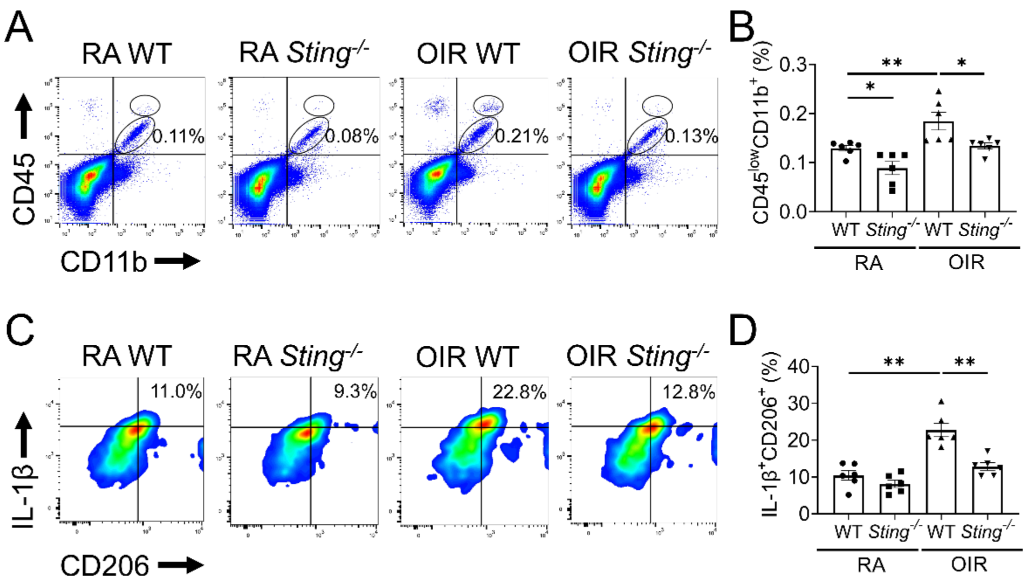

Figure S4

Figure S4. Knockout of *Sting* attenuated the overactivation of retinal microglia cells in the OIR model. A: Representative flow cytometric plots of retinal microglia cells ($CD45^{low}CD11b^{+}$) in the retinas of WT and *Sting*$^{-/-}$ mice in RA control and OIR groups at P17. The lower and upper ellipse-shaped gates indicated $CD45^{low}CD11b^{+}$ and $CD45^{high}CD11b^{+}$ cells, respectively. B: Flow cytometric quantification of retinal microglia percentage in retinal cells in (A) (n = 6). C:

Representative flow cytometric plots of IL-1β^+CD206$^+$ in CD45^{low}CD11b$^+$ cells in the retinas of RA controls and OIR mice at P17. D: Flow cytometric quantification of IL-1β^+CD206$^+$ fractions in retinal CD45$^+$CD11b$^+$ cells in (C) (n = 6). Data were presented as mean ± SEM. * $P < 0.05$, ** $P < 0.01$.

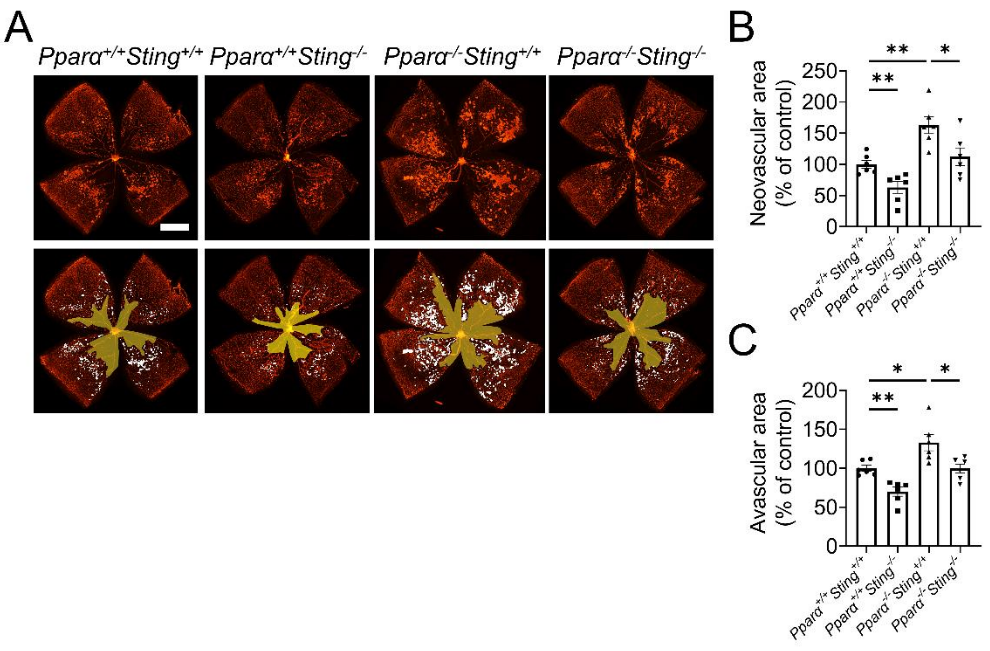

Figure S5

Figure S5. Effect of *Sting* knockout and *Pparα* knockout on retinal pathological angiogenesis in the OIR model. A: Representative images of isolectin-stained retinal flatmounts from WT, *Sting*$^{-/-}$, *Pparα*$^{-/-}$, and *Pparα*$^{-/-}$*Sting*$^{-/-}$ mice with OIR at P17. Scale bar: 1 mm. The neovascular areas and avascular areas were labeled with white color and yellow color, respectively.

B&C: The quantification of neovascular areas and avascular areas in (A) ($n = 6$). Data were presented as mean ± SEM. * $P < 0.05$, ** $P < 0.01$.

Table S1. The list of primers used in this study.

mt-Co1 forward	5'…TCGGAGCCCCAGATATAGCA…3'
mt-Co1 reverse	5'…TTTCCGGCTAGAGGTGGGTA…3'
Actb forward	5'…ACCTTCTACAATGAGCTGCG…3'
Actb reverse	5'…CTGGATGGCTACGTACATGG…3'
Mb21d1 forward	5'…ACGGGAGTCGGAGTTCAAAG…3'
Mb21d1 reverse	5'…ATGACTCAGCGGATTTCCTCG…3'
Tmem173 forward	5'…GGCTGGCCTGGTCATACTAC…3'
Tmem173 reverse	5'…GTACAGTCTTCGGCTCCCTG…3'
Il1β forward	5'…TGCCACCTTTTGACAGTGATG…3'
Il1β reverse	5'…ATGTGCTGCTGCGAGATTTG…3'
Tnfα forward	5'…GACAAGCCTGTAGCCCACG…3'
Tnfα reverse	5'…CCTTGAAGAGAACCTGGGAGT…3'
Vegf forward	5'…CTGGACCCTGGCTTTACTGC…3'
Vegf reverse	5'…CTGCTCTCCTTCTGTCGTGG…3'
Ccl2 forward	5'…CTGCTGTTCACAGTTGCCG…3'
Ccl2 reverse	5'…GCACAGACCTCTCTCTTGAGC…3'
Emr1 forward	5'…CCCTGGGACAAACACTTGGT…3'
Emr1 reverse	5'…TTGACATTCCACTCCTGGGC…3'
Ifnb1 forward	5'…TGGGAGATGTCCTCAACTGC…3'
Ifnb1 reverse	5'…CCTGCAACCACCACTCATTC…3'

64 **Table S2. The list of antibodies used in this study.**

Antibodies	Host	Dilution	Company	Catalog No.
cGAS	Rabbit	1:1000	Cell signaling	31659
STING	Rabbit	1:1000	Proteintech	19851-1-AP
CD11b	Rat	1:100	ThermoFisher	14-0112-82
CD31	Goat	1:200	R&D Systems	AF3628
VEGF	Mouse	1:500	Santa Cruz	sc-7269
Albumin	Goat	1:1000	Bethyl Laboratories	A90-134A
Anti-rabbit IgG	Goat	1:3000	Vector Laboratories	PI-1000
Anti-mouse IgG	Horse	1:3000	Vector Laboratories	PI-2000
Anti-goat IgG	Donkey	1:3000	Vector Laboratories	PI-9500
Alexa-700 anti-CD11b	Rat	1:100	Biolegend	101222
APC anti-F4/80	Rat	1:100	Biolegend	123116
PE anti-CD45	Mouse	1:100	Biolegend	157604
APC-eFluor780 anti-IL1β	Rat	1:100	eBioscience	47-7114-82
FITC anti-CD206	Rat	1:100	Biolegend	141704
Fc block	Rat	1:50	BD Biosciences	553141

65
